# Bedside detection of end-tidal overdistention: an exploratory comparison of loading maneuvers

**DOI:** 10.1186/s40635-025-00842-9

**Published:** 2026-01-19

**Authors:** Rebecca L. Kummer, Lauren T. Thornton, John E. Selickman, Firas S. Elmufdi, Philip S. Crooke, John J. Marini

**Affiliations:** 1 Division of Pulmonary, Allergy, Critical Care and Sleep Medicine, University of Minnesota, Minneapolis, MN USA; 2 Respiratory Consultants, Robbinsdale, MN USA; 3 Department of Pulmonary and Critical Care Medicine, Regions Hospital, MS11203B, 630 Jackson Street, St. Paul, MN USA; 4 Department of Mathematics, Vanderbilt University, Nashville, TN USA

**Keywords:** Acute respiratory distress syndrome, Covid-19, Mechanical ventilation, Tidal overdistention, Chest wall compression

## Abstract

**Background:**

Paradoxical improvement in respiratory system compliance with chest wall loading (‘mechanical paradox’) has been well described in adult respiratory distress syndrome (ARDS), especially in the setting of severe Covid-19 pneumonia. A standardized bedside technique of chest wall loading to elicit this paradoxical response has not been fully developed.

**Methods:**

In two community ICUs, adult patients who were passively ventilated with volume control for diverse conditions underwent a series of stepwise compression maneuvers: first, manual compressions of the chest and abdomen in the semi-Fowler and supine positions; then, compressions of the chest and abdomen with 2, 6, and 10 kg saline bags in the supine position. These maneuvers were conducted with small and large surface ‘footprints’. Under each loading condition, three breath cycles were allowed to pass before tidal volume, PEEP, peak, and plateau pressures were recorded.

**Results:**

Ten patients were included in the case series. Only one of ten patients demonstrated mechanical paradox, which was elicited both by chest wall loading and by moving the patient from the semi-Fowler to the horizontal position. In all patients, abdominal compression elicited a larger change in plateau pressure than did sternal compression. At least 6 kg of weighting force was needed to detect a meaningful change in plateau pressure.

**Conclusions:**

Mechanical paradox occurs infrequently outside of very severe, unresolving ARDS. Apart from compression over the upper abdomen, a simple bedside maneuver for detection of mechanical paradox may be moving the patient from the semi-upright to the supine position.

## Background

Lung protective ventilation has been a cornerstone of ARDS management for over two decades[1]. Smaller than traditional tidal volumes recognize the reduced functional “baby lung” capacity of ARDS, in which well-aerated units make up only a small portion of the total lung tissue—often approximating the functional volume of a 5-year-old’s lungs [2]. Clinicians strive to maintain plateau and driving pressures below their generally accepted “safe” upper limits and thereby minimize the risk of excessively stretching the baby lung and promoting ventilator-induced lung injury (VILI). Even when operating within these safety guidelines, however, certain sectors of the baby lung may over-distend at the end of each tidal inflation, especially as respiratory system compliance (Crs) declines.

**Fig. 1:**
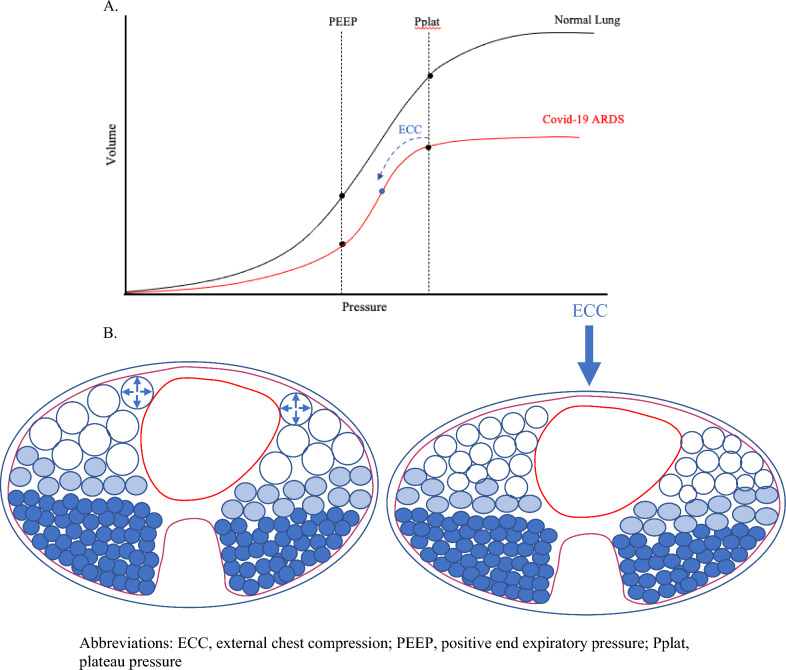
**A** In the low compliance state of Covid-19 ARDS, chest wall loading likely compresses the overdistended lung units, decreasing the volume of the baby lung and allowing it to operate on the linear portion of the pressure volume curve, improving tidal compliance. **B** Cross-sectional diagram of the chest at end-inspiration illustrating the alveolar overdistention of aerated baby lung units (large anterior open blue circles), which is relieved with external chest compression (ECC). ECC: external chest compression; PEEP: positive end-expiratory pressure; Pplat: plateau pressure

Several practical methods for detecting end-tidal overdistention have been described. Traditionally, PEEP titration or examination of the contour of the pressure volume curve has been used for this purpose [3, 4]. Both may be time consuming and require manipulation of ventilator settings. More recently, noting the directional response of respiratory system tidal compliance to transient chest wall loading has gained attention. During the Covid-19 pandemic, numerous reports called attention to the paradoxical *improvement* in respiratory system tidal compliance during chest wall loading [5–8]. When disease-free, external loading of the chest wall is expected to increase plateau and driving pressures due to reductions in chest wall compliance and lung derecruitment. However, in severe ARDS, external compression may allow overdistended lung units to operate at lower *transpulmonary* pressures, improving tidal compliance of both lung and respiratory system (Fig. 1) [6, 9]. We note that in non-overdistended patients, chest wall loading may reduce resting *lung* volume; however, such reductions of FRC do not simultaneously improve the tidal compliance of the integrated *respiratory system* when PEEP and VT remain unaltered. By contrast, an increase of PEEP or of VT does have the potential to worsen tidal compliance when external loads are applied.

In a prospective physiologic study by Batista and colleagues, external chest-wall compression with a 5-L saline bag applied across the sternum of adult patients with Covid-19 ARDS significantly improved respiratory system compliance at 5 and 30 min due to significantly enhanced tidal lung compliance (Clung). Using electrical impedance tomography, they showed that improved ventilation predominated in the non-dependent portions of the lung, suggesting less hyperinflation of these areas during chest-wall compression, as opposed to increased recruitment in dependent lung zones [10]. Interestingly, the benefits of external chest-wall compression on Crs and driving pressure were most notable at 5 min of compression and faded as compression continued. The authors concluded that chest-wall loading may be a useful diagnostic maneuver but that currently there is no evidence for its sustained benefit in the treatment of ARDS [10].

Diagnostic chest wall loading appears to be safe, universally available, and quickly applied and reversed. However, no standardized and quantitatively validated method currently exists for performing this bedside maneuver. With this in mind, the primary aim of our study was to develop a reproducible bedside technique to detect mechanical paradox for intubated, passively ventilated patients with and without pulmonary disease in the intensive care unit (ICU).

## Methods

The design of this trial was a prospective case series testing a physiologic response to a physical intervention. The study took place in the medical intensive care units of two University of Minnesota-affiliated community hospitals in Minneapolis/St. Paul (USA). Enrollment occurred between August 2023 and August 2024. Adult patients admitted to the ICU who were intubated and passively ventilated were eligible for inclusion. Passively ventilated was defined as receiving deep sedation sufficient to suppress any active breathing (Richmond agitation and sedation score ≤ − 3 and measured respiratory rate equal to set respiratory rate without evidence of patient-triggered breaths), or chemical neuromuscular blockade received within the prior 30 min and no visual or ventilator evidence of breathing effort, or presence of an unremitting neurologic injury that prevented active breathing at baseline. Exclusion criteria were: age less than 18 years; not passively ventilated; hemodynamic instability (based on clinical assessment of the research team in conjunction with the primary treatment team); pregnancy; prone position; chest or abdominal trauma or surgery within the prior 30 days; traumatic brain injury (TBI), intracranial hemorrhage, or neurologic procedure within the prior 30 days; or inability to obtain informed consent. This trial was approved by the HealthPartners Institutional Review Board that serves both study hospitals.

The ICU census at each community hospital was reviewed daily (Monday–Friday) by a member of the research team. All patients receiving mechanical ventilation were screened for eligibility. If passivity on the ventilator could not be determined through chart review, the patient was assessed directly at the bedside by a member of the research team before enrollment. If a patient was deemed eligible for the study, the patient’s surrogate decision-maker was contacted for informed consent. After enrollment, the patient’s baseline characteristics were extracted from the electronic medical record (EMR), and vital signs, ventilator settings, and respiratory mechanics were recorded. If not already supported by volume-targeted assist control ventilation, the patient was switched to this mode of ventilation for the study period. Plateau pressure was obtained by performing an inspiratory hold maneuver, and the average value for three such determinations was calculated and recorded. An expiratory hold maneuver was performed to quantify intrinsic PEEP, if present. Ventilator settings—including PEEP—were left unchanged during these interventions.

**Fig. 2:**
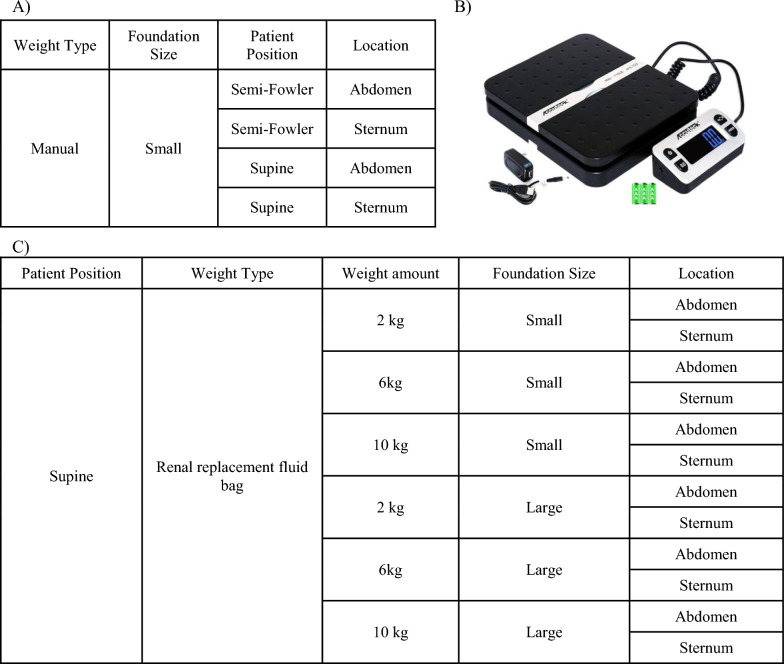
Compared compression maneuvers. **A** Manual compressions were performed over the ‘small’ scale foundation, first with the patient in semi-Fowler position over the abdomen, then over the sternum; next in the supine position over the abdomen then the sternum. **B** The scale used for quantifying manual force as weight in kg and as the small foundation. **C** Compression maneuvers with saline bags were performed with the patient in supine position, first with a small foundation, moving through the 2 then 6 then 10 kg weights over the abdomen then sternum; next with a large foundation moving through the 2 then 6 then 10 kg weights over the abdomen then sternum

**Fig. 3:**
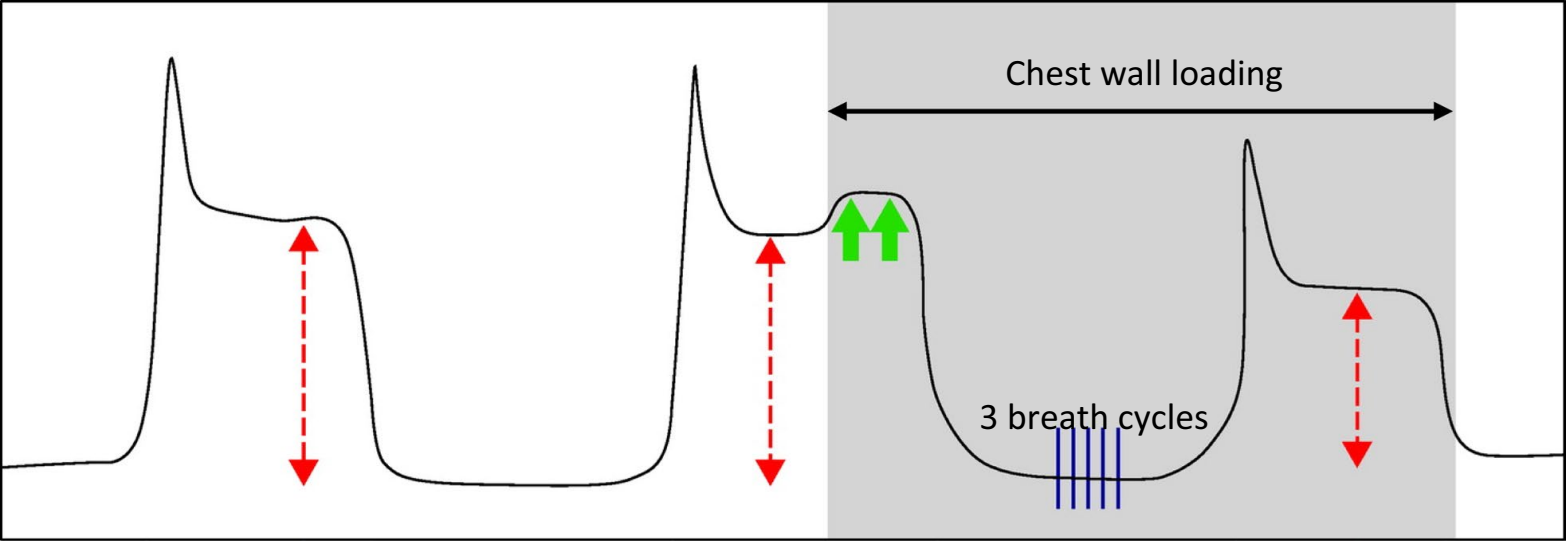
Determining manual force (measured in weight) required to detect mechanical paradox. Adapted with permission from Dr John Marini (PMID 35849421). Left waveform: end-inspiratory hold without chest wall loading, for comparison. Middle waveform: end-inspiratory hold with sufficient pressure applied either to sternum or abdomen (shaded zone bounded by horizontal arrowheads) to briefly deflect the plateau pressure upward by 2 cmH2O (green arrows). This pressure (measured in weight by the scale) was recorded and then sustained over three breath cycles (blue dashed lines). Right waveform: an end-inspiratory hold is repeated after chest wall loading for 3 tidal cycles and the new airway pressures are recorded under the load, indicating a significant paradox response to loading

Chest wall compression maneuvers were then performed in a stepwise fashion (Fig. 2). For manual compression, a 17 by 20 by 3.8 cm flat electronic strain gauge scale (ACCUTECK→) was placed on the patient’s chest or upper abdomen, and enough pressure (measured as weight in kg) was applied manually to increase the airway pressure by 2 cmH_2_O during an end-inspiratory occlusive maneuver (Fig. 3); this weight equivalent was then recorded at each site. (Although arbitrary, the value of 2 cmH2O is readily observable, feasible to safely attain, and has been used in previous reports [5, 8].) The inspiratory hold was released while maintaining a stable application of weight over the scale, and three breaths were allowed to cycle. For the fourth breath, peak airway pressure, plateau pressure (measured with an inspiratory hold), PEEP, and tidal volume were recorded (load data). Manual pressure was then released, three breath cycles were allowed to pass, and the same variables were again recorded (release data).

All non-manual interventions were performed with the patient in the supine-horizontal position. For weighted measurements, continuous renal replacement fluid bags were applied to either the chest or abdomen, and for each condition, 3 breathing cycles were allowed to pass before data collection. Weights of 2 kg, 6 kg, and 10 kg were chosen a priori. For the 2 kg weight, a 5-L renal replacement fluid bag was drained down to 2 kg so that the weight would be distributed over the same surface area. For the 6 kg weight, a 1L saline bag was applied on top of the 5-L renal replacement bag, and for the 10 kg weight, two 5-L fluid bags were used, one stacked upon the other. The renal replacement bags served as the “large” footprint. The 17 × 20 cm strain gauge scale served as the “small” footprint with the saline bags balanced on top. The weight of the scale itself was < 1 kg and was considered negligible for the purposes of our study. Following the termination of data collection, participants were returned to the pre-intervention ventilator mode when applicable.

Safety precautions during data collection included: (1) continuous monitoring of blood pressure via arterial catheter or via blood pressure cuff every 3 min and commitment to terminate the study if mean arterial pressure (MAP) fell ≥ 10 mmHg or below the caregiver team’s stated goal (generally < 65 mmHg); (2) continuously monitoring of heart rate and commitment to terminate the study if the heart rate rose by > 15 bpm or an arrhythmia developed; (3) continuous monitoring of tissue oxygenation via pulse oximetry, terminating the study if the FiO2 had to be increased to 1.0 to maintain SpO2 ≥ 88%; (4) monitoring of airway pressures after any intervention or change in ventilator settings and terminating the study if plateau pressure rose and sustained above the clinically acceptable threshold of 30 cmH_2_O during a loading maneuver.

An enrollment size of 30 patients was planned to compare estimates of mechanical properties among techniques (half-width of 95% CI) ranging from 0.19 (0.5 incidence) to 0.12 (0.1 or 0.9 incidence) and to provide 80% power to detect predictors of mechanical paradox with large effect sizes (Cohen’s *h* = 1.0 to 1.2 for predictor level ratios of 1:1 to 3:1) at the 2-sided 5% level of significance. A paired t-test was used to compare the effect of 2, 6, and 10 kg weights applied to the abdomen and sternum on change in plateau pressure. An ANOVA analysis was used to compare the 6 kg weight against the 2 kg and 10 kg weights at the abdomen and sternum.

## Results

A total of 10 patients who qualified were enrolled consecutively during the study period. Data acquisition was stopped short of the planned total of 30 patients due to slow enrollment (the majority of the study was conducted after cessation of the Covid-19 pandemic). The demographics and baseline characteristics of the enrolled patients are shown in Table 1. The average age was 55 years, half were female, and the average BMI was 37.7 kg/m^2^. Of note, all patients had been hospitalized for one to seven days at the time of the study (i.e., during the first week of hospitalization) with the exception of patient #1, who was enrolled on hospital day 17. Two patients, #1 and #7, tested positive for Covid-19.

**Table 1:** Patient demographics and baseline characteristics

Patient	Age (yr)	Gender	PBW (kg)	BMI (kg/m^2^)	Hospital LOS (d)	MV duration (hr)	Pulmonary disease	P/F ratio	NMB	Baseline Pplat (cmH2O)	Set PEEP (cmH2O)	Paradox?
1	77	F	52.4	27.5	17	21	Covid-19 ARDS	108	Yes	31	10	Yes
2	53	M	82.2	23.8	3	72	Pneumonia	566	No	13	5	No
3	37	F	57	53.9	1	11	Asthma exacerbation	167	Yes	25	7	No
4	62	M	82.2	22.1	1	11	None	406	No	13	5	No
5	45	F	57	53.4	2	12	Non-Covid-19 ARDS	238	No	25	10	No
6	55	M	59.2	34.5	7	40	Non-Covid-19 ARDS	130	Yes	24	7	No
7	60	F	57	23.3	5	18	Covid-19 ARDS	57	Yes	23	12	No
8	56	M	66	23.8	1	50	None	250 (S/F)	No	17	5	No
9	47	M	80	59.3	4	78	Asthma exacerbation	282	No	21	10	No
10	55	F	55.6	55.6	1	24	Non-Covid-19 ARDS	101	Yes	24	12	No

PWB: predicted body weight; BMI: body mass index; LOS: length of stay; MV: mechanical ventilation; P/F: PaO_2_/FiO_2_; NMB: neuromuscular blockade; Pplat: plateau pressure; PEEP: positive end-expiratory pressure

Mechanical paradox was observed only in patient #1. The pressure and volume data with abdomen and chest wall loading for patient #1 are displayed in Table 2. Maneuvers were performed in the order in which they are documented. With the patient in semi-Fowler position, manual loading of the abdomen and chest wall dropped the plateau and driving pressures by 5 cm H_2_O. Moving the patient to supine position from semi-Fowler position for the remainder of the data collection using chest or abdominal weighting decreased the plateau pressure from 29–30 cmH2O to 24–25 cmH2O.

**Table 2:** Pressure and volume data with abdomen and chest wall loading for patient #1 with mechanical paradox

Position	Pressure type	Pressure location	Foundation size	Load or release	Peak pressure	Plateau pressure	Change in plateau pressure
Semi-Fowler	Manual	Abdomen	Small	Load	26	24	
Semi-Fowler	Manual	Abdomen	Small	Release	33	29	− 5
Semi-Fowler	Manual	Sternum	Small	Load	27	25	
Semi-Fowler	Manual	Sternum	Small	Release	33	30	− 5

There is a significant reduction in plateau pressure with manual loading while the patient is in the semi-Fowler position. The decrease in plateau pressure with loading is more modest in the supine position

Patients 2–10 were ventilated with plateau pressures <  = 25 cmH2O and did not demonstrate mechanical paradox. For these patients, the average changes in plateau pressure by maneuver type are shown in Table 3. For two patients within this cohort (#7 and 8), loading the abdomen with 10 kg of weight increased and sustained the plateau pressure above the safety cutoff of 30 cmH_2_O; consequently, the weight was removed and data were not collected for this intervention. No other safety violations occurred in any patient. Results of a paired student’s t-test comparing the ‘weighted’ data (excluding the manual data) revealed that applying pressure over the abdomen as compared to the sternum achieved a statistically significant, larger change in plateau pressure (mean difference 0.61 cmH_2_O, *p* value = 0.013, 95% CI 0.134–1.082). Comparing compression of the abdomen vs sternum across each non-manual weight (2, 6, and 10 kg), only the 6 kg weight had a statistically significant larger rise in plateau pressure with compression of the abdomen (mean difference 1.06 cm H_2_O, *p* value = 0.006, 95% CI 0.342–1.77), whereas the 2 kg and 10 kg weights did not (mean difference 0.33 cm H_2_O, *p* value = 0.163, 95% CI − 0.149 to 0.816 and mean difference 0.4 cm H_2_O, *p* value = 0.541, 95% CI 0.969–1.77, respectively).

**Table 3:** Average △ Pplat by maneuver type in patients without mechanical paradox (*n* = 9)

Maneuver	Man, SF, Ab	Man, SF, St	2 kg, Sm, Ab	2 kg, Sm, St	6 kg, Sm, Ab	6 kg, Sm, St	10 kg, Sm, Ab	10 kg, Sm, St
Average △ Pplat	1.67	1.44	0.67	0.44	2.56	1.56	3.43	3.11
Maneuver	Man, Sup, Ab	Man, Sup, St	2 kg, Lg, Ab	2 kg, Lg, St	6 kg, Lg, Ab	6 kg, Lg, St	10 kg, Lg, Ab	10 kg, Lg, St
Average △Pplat	2.44	1.67	0.78	0.56	1.89	1.22	3.33	2.56

Note that the 10 kg abdominal loading data come from the average of 7, rather than 9, patients due to mentioned safety violation with patient #7 and 8 in that abdominal loading caused the plateau pressure to sustain above 30 cm H_2_O

Ab: abdomen; Lg: large; Man: manual; Pplat: plateau pressure; SF: semi-Fowler; Sm: small; St: sternum; Sup: supine

## Discussion

In this case series, only one out of ten patients demonstrated mechanical paradox, as evidenced by marked decreases of plateau and driving pressure during chest wall loading. While it is difficult to draw any firm conclusion based on a single patient’s dataset, we note that this individual with paradox had been hospitalized with acute hypoxic respiratory failure for longer than 2 weeks at the time of enrollment, whereas all other study subjects had been supported for 1 week or less. As mechanical paradox has been reported primarily among late phase and severe ARDS [5–10] perhaps it is not surprising that we detected it only in the patient who was enrolled two and a half weeks into her respiratory illness. Her respiratory pathology was quite advanced, as reflected by a P/F ratio of 108 and a baseline plateau pressure of ~ 30 cmH_2_O, a value at the upper limit of lung protection guidelines and higher than any other in the sampled cohort. Another distinguishing characteristic is that her severe pneumonia was due to Covid-19. Though reported in other forms of severe lung injury [6], mechanical paradox has been described primarily as a phenomenon consequent to Covid-19 ARDS [5, 7, 8, 11]. Our diverse sample included patients studied within the first week after intubation with non-Covid ARDS, asthma exacerbation, community acquired pneumonia, and no underlying lung pathology; none of these demonstrated mechanical paradox.

It is also worth noting that the mechanical paradox was uncovered by simply transitioning patient #1 from the semi-Fowler to the supine position, a loading maneuver resulting from cephalad displacement of the diaphragm. This maneuver, if sensitive enough, would arguably be simpler than exerting sternal or abdominal pressure as it does not require concern for the amount of force applied over a specific location. Indeed, this phenomenon has been previously described in the setting of Covid-19 ARDS [6, 12–14]. So far, these reports are limited to smaller case series, and larger studies are needed to determine the sensitivity of position change for eliciting the mechanical paradox.

Despite the lack of positive mechanical paradox findings in this case series, several observations we made regarding procedure execution deserve mention. For the nine patients who did not demonstrate mechanical paradox, applying force over the abdomen resulted in a significantly greater change in plateau pressure (and pleural pressure) than applying the same force over the sternum. This difference may owe to the relative rigidity of the thoracic cage as compared to the relatively compliant abdominal wall. The 6 kg weight resulted in the greatest mean difference in plateau pressure between compression of the abdomen vs sternum. We speculate the 2 kg weight was insufficient to elicit a significant change in plateau pressure for the average adult at either location, while 10 kg was enough weight to overcome the outward recoil forces of the chest wall and cause similar lung compression at the chest cage and abdomen. Along the same line, performing loading maneuvers in the supine position resulted in a greater change in plateau pressure as compared to the semi-Fowler position for the same external compressive force, likely because of the added sub-diaphragmatic pressure exerted by the abdominal contents when supine. Distribution of the force over a small vs large surface footprint did not make a difference in terms of change in plateau pressure for the same applied force. In this study, substantial external pressure was needed to detect a meaningful change in plateau pressure. The 2 kg weight did not raise the plateau pressure more than 1 cm H_2_O, and the average manual weight applied to the abdomen to increase plateau pressure by 2 cm H_2_O was 5.9 kg on the abdomen and 7.8 kg on the sternum.

Our experience suggests a simple, quickly reversible method with which a clinician could detect net end-tidal overdistention by eliciting mechanical paradox: tracking plateau and driving pressures while changing the patient from the semi-Fowler to the supine horizontal position or by steady manual application of a firm (6 kg) force over the central abdomen. These interventions do not require additional technology or manipulation of the ventilator, other than ensuring the patient is ventilated passively in the volume control mode. While these may prove less sensitive than esophageal manometry or electrical impedance tomography in detecting end-tidal overdistention [10], they are non-invasive, universally available, and rapidly performed.

This case series has several important limitations, the first of which is the small sample size with only one of ten patients demonstrating mechanical paradox. Our study was terminated prior to the goal enrollment of 30 patients due to the relative paucity of suitable passively ventilated patient candidates in this post-pandemic study. Although the small sample size and diversity of this exploratory exercise make it impossible to draw firm conclusions regarding incidence, our findings strongly imply that the paradoxical response is not universal when lung protective guidelines are observed. Indeed, it may only be elicited when the upper pressure boundary of a ‘lung protective’ plateau is approached or exceeded. We also did not raise PEEP, an intervention that would accentuate any regional hyperinflation that gives rise to paradox. In addition, external saline weights were applied only with the patient in the horizontal orientation, a body position that reduces resting lung volume (FRC) and favors reduction of end-tidal hyperinflation at baseline, prior to external loading.

## Conclusions

Based on the data gathered from this diverse series of passively ventilated patients in the ICU, we conclude that mechanical paradox is unusual outside the setting of severe and unresolving ARDS. Our comparison indicated that either applied over the sternum or upper abdomen, 6 kg of external weight (or its manual equivalent) seems necessary to detect a 2 cmH2O change in plateau pressure during an end-inspiratory pause, an observation that indicates a meaningful rise in pleural pressure and reduction of transpulmonary pressure during tidal ventilation. In applying a given level of chest wall force, abdominal pressure is more efficient than sternal pressure at changing the plateau pressure, and by inference, the average pleural pressure at end inspiration. At a given site, however, the distribution of surface weight (the external force footprint) does not make a major difference to the resulting elevation of these pressures.

## Data Availability

The data collected during the current study are available from the corresponding author upon reasonable request.

## Abbreviations

ANOVA: Analysis of variance
ARDS: Adult respiratory distress syndrome
BMI: Body mass index
Clung: Lung compliance
Crs: Respiratory system compliance
EMR: Electronic medical record
ICU: Intensive care unit
MAP: Mean arterial pressure
PEEP: Positive end-expiratory pressure
TBI: Traumatic brain injury
VILI: Ventilator-induced lung injury
